# Virulence of Tuberculosis in Palestine

**Published:** 1914-10

**Authors:** 


### ABSTRACTS

**Virulence of Tuberculosis in Palestine.** Hans Much, Brit. Jour. of Tuberculosis, January, 1914, states that there are now only a few hundred lepers but hundreds of thousands of tuberculous persons in Palestine, the former not being ostracized but the latter being avoided and often treated cruelly. 95% of persons over ten years of age in Europe, react positively to tuberculin while in Palestine only 25% do so and these mainly in towns. In Europe, tuberculosis is a children's disease and produces more or less immunity, both in the subsequent life of the individual who recovers, and by heredity so that the disease has become mitigated. Palestine is a fresh field for tuberculosis, infected by Russian Jews and returned American emigrants and the disease assumes a virulent type.

